# QUALITATIVE DATA INTEGRATION IN DISASTER PREPAREDNESS CAREGIVER INTERVENTION

**DOI:** 10.1093/geroni/igae098.3416

**Published:** 2024-12-31

**Authors:** Brittany Anderson, Morolake Adeagbo, Maria Donohoe, Tiffany Wunderlich, Sato Ashida, Aaron Seaman

**Affiliations:** University of Iowa, Iowa City, Iowa, United States; University of Iowa, Iowa City, Iowa, United States; University of Iowa, Iowa City, Iowa, United States; University of Iowa, Iowa City, Iowa, United States; University of Iowa, Iowa City, Iowa, United States; University of Iowa, Iowa CIty, Iowa, United States

## Abstract

Disaster PrepWise Caregiving (DPW-C) is an online disaster preparedness program for caregivers of persons with dementia (PWD). DPW-C works with collegiate AmeriCorps volunteers to develop and build caregivers’ household disaster plan in a location of their choosing, whether at home, in public, or through online platforms. The program utilizes an all-hazards approach, which highlights all-encompassing disaster preparedness, while accommodating the specific needs of caregivers and the importance of maintaining their caregiving tasks during and after a disaster. Within the hybrid type one trial, an evaluation of the program’s potential determinants of implementation is being conducted. Guided by the PRISM (Practical, Robust Implementation, and Sustainability Model) framework, the team is utilizing the triangulation of qualitative methods to assess the implementation process and fidelity through observational evaluation of the social interactions between the AmeriCorps program interventionists and the caregiver participants. This entails examining interventionists’ communication and engagement with caregivers regarding their disaster plans. Observation data elucidate traits and themes in approach and engagement that were more successful in leading to positive social interactions that facilitated in-depth discussion and careful development of the household disaster plan. In response to the project’s needs, the research team is using observation data to adapt the program and intervention processes to improve the overall success of interventions. As the project progresses, the qualitative team will expand the project evaluation by including examinations of the program’s effectiveness and long-term sustainability. This poster will discuss how the qualitative team helped integrate gained knowledge to improve the program implementation.

